# Development of an Interprofessional Education Project in Dentistry Based on the Positive Behavior Support Theory: Pilot Curriculum Development and Validation Study

**DOI:** 10.2196/50389

**Published:** 2024-11-11

**Authors:** MengWei Pang, WeiYu Lu, Chuling Huang, Meixiu Lin, Jiangsheng Ran, Xiaomei Tang, YuanDing Huang, Sheng Yang, Jinlin Song

**Affiliations:** 1 College of Stomatology Chongqing Medical University Chongqing China; 2 Chongqing Key Laboratory of Oral Diseases Chongqing Medical University Chongqing China; 3 Chongqing Municipal Key Laboratory of Oral Biomedical Engineering of Higher Education Chongqing Medical University Chongqing China

**Keywords:** innovative interprofessional education, dentistry, dental technology, positive behavior support, IPE, positive behavior, training system, dental education

## Abstract

**Background:**

Effective interprofessional education (IPE) can facilitate teamwork between dentists and dental technicians, thereby enabling the efficient provision of high-quality dental care.

**Objective:**

This study aimed to design and assess an IPE module named Project 35, which was offered to dental and dental technology students early in their undergraduate training as a precursor to a more comprehensive IPE curriculum in dentistry and dental technology.

**Methods:**

Leveraging positive behavior support (PBS) theory, Project 35 was devised as an innovation and entrepreneurship educational training framework. It used project-based learning to cultivate teamwork skills and to promote the professional development of dental and dental technology students. The pilot study was designed to present the IPE module and preliminarily assess its validity. In survey 1, which was conducted immediately after the course, the dental and dental technology students’ self-reported skill acquisition and attitudes were assessed and compared. Survey 2, conducted 1 year after the course, focused on the comparative benefits of Project 35 training for dental technology students versus an untrained group.

**Results:**

A total of 66 students, including 36 dental students and 30 dental technology students who had undertaken the training, were recruited. Project 35 training improved teamwork skills for students in both disciplines comparably, and the students recognized the training as highly valuable and effective. The mean values for all items indicating skills improvement of students ranged from 4.13 (SD 0.797) to 4.63 (SD 0.495) for dental students and from 4.13 (SD 0.869) to 4.74 (SD 0.619) for dental technology students. Among the dental technology students, the trained group showed greater independent and innovative approaches and was more optimistic about the future of the profession than the nontrained group (*P*<.05).

**Conclusions:**

Despite the small sample size, the validity of the Project 35 training system was evident, and the success of our pilot study provides a sound basis for the future development of IPE in clinical dental and dental technology education programs.

## Introduction

The provision of a successful oral prosthesis requires effective teamwork between the dentist and the dental technician through accurate work authorization, timely and productive feedback, and good coordination [1-3]. Excellent teamwork skills are essential for dentists and dental technicians for efficient clinical practice [4]. However, inadequate teamwork between dentists and dental technicians frequently occurs and may include flawed assumptions about the responsibilities for prosthesis design or deficient feedback [5]. Many contemporary dental curricula lack the specific teaching of teamwork and communication skills, which could translate to a failure of collaboration between dentists and dental technicians, ultimately contributing to treatment failures, unnecessary delays, and losses; these developments can lead to mutual accusations and disputes [6]. Teamwork skills can be pedagogically taught and learned. Dental and dental technology students can be helped to develop teamwork skills and improve their partnership through a well-designed interprofessional educational curriculum [7,8], which eventually benefits all stakeholders in dental practice [9]. Basic educational theory, curriculum content selection, teaching and learning approaches, and entry timing of interprofessional education (IPE) in dentistry and dental technology are key focus areas in this research field [10,11].

Positive behavior support (PBS) is an established method of providing positive feedback to difficult or challenging groups of trainees. In IPE, using PBS may serve to ensure that groups in danger of being devalued are helped to assume valued social roles, thereby increasing the likelihood that they will be accorded respect from others and receive an unbiased assessment of their contributions within the team [12,13]. Recent studies have confirmed the benefits of applying PBS in improving the skills and knowledge of medical care teams [14-16], although research related to the application and efficacy of PBS in higher education and IPE is limited. Since there is often a tendency to devalue the contributions of others in dental care teams in practice [3], it seems appropriate to apply PBS as the basic educational theory in IPE for dental care teams.

The behavioral nature of the interactions between dentists and dental technicians can be summarized as commercial behavior under a collaborative service delivery model [13]. To simulate this behavior in early education, well-coordinated IPE curricula for dentistry and dental technology should include innovation and entrepreneurship education content. In medical education, the innovation and entrepreneurship curriculum has been implemented extensively. Program design places a strong emphasis on collaboration and leadership [17]. The majority of students in a Chinese study report being happy with their programs, believing them to be helpful in expanding their professional horizons and improving their strong practical components [18,19]. Notably, dental and dental technology graduates are likely to be entrepreneurs starting their own dental clinic or dental laboratory [20]. In this type of career, entrepreneurial skills and product or process innovations are proven and powerful means to promote survival in the competitive dental market [21]. Innovation and entrepreneurship [22] curricula may be of great value and are necessary to include in IPE for dentistry.

Some studies suggest that improving teamwork skills through IPE should occur early in the education of health care professionals, while others take the opposite view, considering a thorough knowledge of one’s own discipline as essential to understanding the contributions required for successful teamwork [23]. To bridge this difference in perspective, project-based learning (PBL), a commonly applied learning approach in innovation and entrepreneurship education, appears to be valuable. PBL is an active student-centered form of instruction that is characterized by students’ autonomy, constructive investigations, goal setting, collaboration, communication, and reflection within real-world practices [24]. This framework provides students with early exposure to real clinical practice scenarios and allows for the application of technical skills in various domains, including the development of new technologies. It also facilitates a basic understanding of key issues related to technology commercialization [25]. PBL is an effective approach in innovation and entrepreneurship courses in engineering and computer science education [24,26-28], albeit relatively few studies have addressed PBL and innovation and entrepreneurship curricula for medical and health care education [29]. Considering the rapid growth of engineering and computational content within dental curricula, PBL may be similarly beneficial for dentistry and dental technology IPE.

In this study, we designed an innovation and entrepreneurship training module named “Project 35”, which is built on PBL principles and PBS theory and was offered to dental and dental technology students early in their undergraduate training as a precursor to a more comprehensive IPE curriculum in dentistry and dental technology. After 4 years of application of this training module, subsets of trained and untrained students were selected to determine the validity of the training module and to inform the development of a formal curriculum.

## Methods

### Curriculum Intervention Design Based on the PBS Matrix

**Figure 1:**
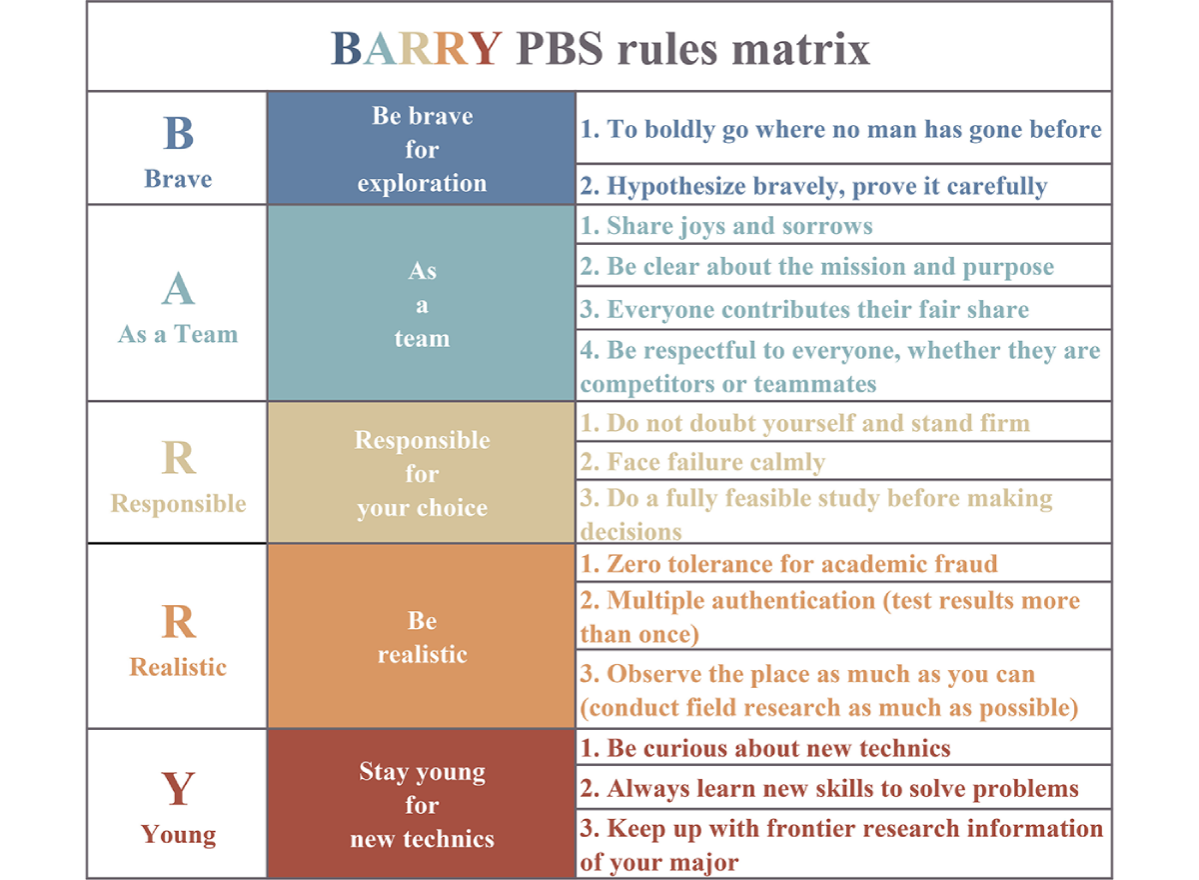
The positive behavior support rules matrix applicable to Project 35 (Each color represents a fundamental principle and its corresponding behavioral norms).

Based on the PBS theory matrix (Figure 1), the guidelines for action were designed with the BARRY (Brave, As a team, Responsible, Realistic, and Young) PBS rules matrix as the core of interprofessional practice and communication. The system actively used interdisciplinary elements to design the training project, named Project 35. It requires the students to form groups with the help of their instructors, determine their project topic with teammates from another major (which usually requires that it must be related to dental care), and complete the project through a series of tasks such as literature review, fieldwork, and laboratory work, culminating in a project presentation. Throughout the whole process, the BARRY PBS rules matrix could instruct students on how to engage in interprofessional practice and communication, teach themselves new techniques, receive feedback and learn from each other, and, in the end, complete a project.

**Figure 2:**
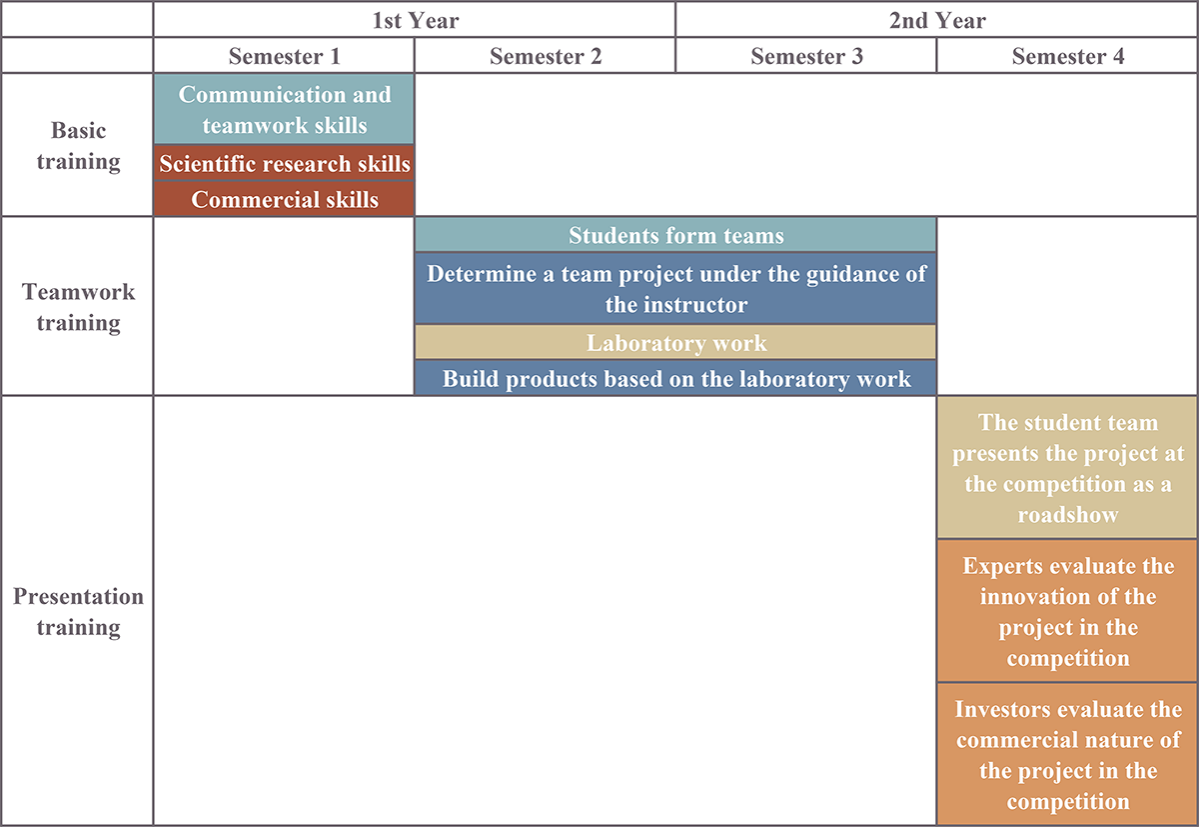
Design and curricular structure of the Project 35 module (Rules in BARRY PBS match with the curriculum in Project 35, consistent with the color expression).

Project 35 was implemented as a 3-phase extracurricular module for first-year dental and dental technology students, with a training cycle of 2 years. All students participated voluntarily and signed an informed consent. In the first phase, the students were divided into groups through simulated recruitment [30] and participated in team-building exercises under the guidance of instructors. After that, all students took theoretical courses based on innovation research theory and commercial theory. Mixed teams ensured that each team consisted of students from both disciplines. In the second phase, microresearch topics designed by the instructor were assigned (Multimedia Appendix 1). The students identified relevant problem areas and completed innovation training under the guidance of the instructor. Students and instructors met biweekly online to discuss project progress. This phase usually took more than 1 year. In the third phase, the innovation designs created in the second phase were packaged as a commercial project, which simulated entrepreneurial training and was carried out in the “sandbox style” [31] through an innovation and entrepreneurship competition. Dental researchers served as judges who evaluated the innovative value of each project. In addition, commercial investors judged the investment valuation of the projects (Figure 2).

### Study Design

#### Participant Recruitment Process

At the initiation of Project 35, first-year dental and dental technology students from the School of Stomatology, Chongqing Medical University, were invited to participate in the training module. The recruitment notice written in Chinese was distributed in class web-based groups, and students who were interested in Project 35 signed up for it by sending an email to the assigned email address. The response emails were expected to include a personal resume of themselves. The complete recruitment procedure is presented in Multimedia Appendix 2. The principal investigator was notified, and a training schedule was determined.

#### Survey 1: Conducted Immediately After the Course

##### Overview

We addressed and compared the validity of Project 35 for dental and dental technology students. A questionnaire administered 1 month after the training was designed to assess the students’ attitudes toward Project 35, and their improvement in relevant abilities was self-reported.

##### Questionnaires’ Design

The questionnaire (Multimedia Appendix 3) was modelled by us on the concept of the “Readiness for Interprofessional Learning Scale” [32,33]. After the pretest and modification, 15 items were determined. A total of 12 items related to the students’ perception of innovation and entrepreneurship training, skill development in communication, and empathy were included, and these items were evaluated using a 5-point Likert scale. Three items collected personal information of the students (including name, grades, and majors), the duration of participation, and their descriptions of what they gained from this training.

#### Survey 2: Conducted 1 Year After the Course

##### Overview

We addressed the question of whether the Project 35 training conferred long-term benefits for dental technology students. In total, 11 out of the 30 dental technology student participants were from the same class, which had a total number of 29 students and were assigned to the training group. Thus, 18 students had not been trained using Project 35 and formed the control group, also called the nontraining group. A second questionnaire was given to all students in this class one year after graduation.

##### Questionnaires’ Design

We designed this questionnaire (Multimedia Appendix 4) to collect the students’ perceptions of innovation and entrepreneurship education and their self-assessed abilities. This preliminary questionnaire was piloted using 5 students who did not participate in the main study to ensure the clarity of the questionnaire [34,35]. Based on their feedback, a panel discussion was organized by the principal investigator to revise some ambiguous words that might cause misunderstanding, to avoid unclear question items, and to design clear and focused questions. Finally, 14 items were ascertained, among which 10 items used a 5-point Likert scale ranging from 1 (strongly disagree) to 5 (strongly agree). In addition, 4 items were used to collect personal information, including participation in Project 35 training and the type of work currently performed by the participant. The remaining 10 items included 4 items that addressed self-evaluation of individual ability, 4 items that addressed self-evaluation of teamwork, and 2 items that addressed professional identity perception. Their academic performance from their school years during the study period was gathered to assess capacity enhancement and evaluate individual ability. These grades included academic grades in professional theory and laboratory courses as well as capability grades that determined their suitability to work in a dental health care team.

#### One-on-One Email Interview

Following questionnaire data collection, one-on-one interviews were conducted by email to capture the students’ subjective perspectives. The emails, with the same content in Chinese, were sent to every participant to reduce bias. The questions in the interview (Multimedia Appendix 5) were sent to each student from the same dental technology class, and they were encouraged to share their feelings and opinions in emails answered. Some representative answers are selected as the sample for keyword extraction [7]. The themes and keywords were manually determined and extracted. Then, the corresponding synonyms, near-synonyms, and antonyms for the listed keywords were used to evaluate participants’ inclinations of the training. Qualitative analyses were conducted using those keywords in the answers.

### Data Analysis

Data were analyzed using IBM SPSS (version 25.0.0.0). For analysis of the questionnaire responses from the dental and dental technology students, the independent-sample *t* test was used. To compare the outcomes of the dental technology students from the training and nontraining groups, the Mann-Whitney *U* rank sum test was applied for non-normally distributed data, and the independent-sample *t* test was applied for normally distributed data. A 2-sided *P*<.05 was regarded as statistically significant.

### Ethical Considerations

This study protocol was reviewed and approved by the Research Ethics Committee of The Affiliated Hospital of Stomatology, Chongqing Medical University (CQHS-REC-2022-LSNo.030; Multimedia Appendix 6). An informed consent form written in Chinese was also sent by email requesting the students’ permission to provide their personal information and academic performance during school years. Students’ information was confidential and anonymous when relevant data were analyzed in this study. The CHERRIES (Checklist for Reporting Results of Internet E-Surveys) is presented in Multimedia Appendix 7.

## Results

**Figure 3:**
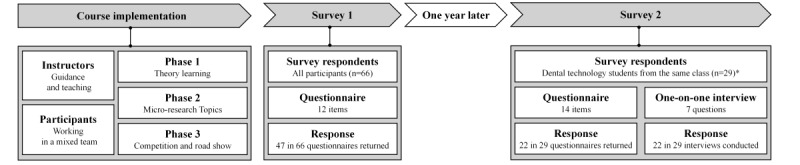
Course implementation and surveys conducted in this study. *11 out of the 30 dental technology student participants were assigned to training group, and they are from the same class consisting 29 students. 18 students who had not been trained using Project 35 formed the nontraining group. All participants in training group and 11 in nontraining group returned the responses and accepted the interview.

Figure 3 shows the workflow of the study.

### Participant Recruitment

A total of 66 students completed the Project 35 training, including 36 dental students and 30 dental technology students. The dental students included in this study accounted for 22.5% (36/160) of the total number of students enrolled in the dental program, and the dental technology students included in this study accounted for 50% (30/60) of the total number of students enrolled in the dental technology program.

### Survey 1: Conducted Immediately After the Course

#### Questionnaires Collection

A total of 47 in 66 questionnaires for participants were returned (response rate=approximately 71%). The questionnaire scores for the dental and dental technology students were summarized and compared (Multimedia Appendix 8). The reliability and validity of the questionnaire had been verified before the analyses (Cronbach α=0.89, Kaiser–Meyer–Olkin value=0.77; Bartlett’s sphericity test: *P*<.001).

#### Questionnaires Analyses

Before enrollment in the Project 35 training, the students’ perceptions of innovation and entrepreneurship were limited, and the mean values of the related item were 2.58 (SD 0.929) and 2.65 (SD 1.071) for dental students and dental technology students, respectively. The mean scores for the rest of the items in the questionnaire were more than 4. Students agreed with their involvement in IPE and knew more about the other majors. After training in Project 35, as Multimedia Appendix 8 showed, students’ motivation for self-improvement was remarkably high, and a markedly positive attitude towards competence enhancement was expressed. Teamwork skills were also improved. Furthermore, the students found the training to be meaningful. There were no significant differences in the scores of the dental and dental technology students (*P*>.05; Multimedia Appendix 8). In the description of their feelings, comprehensive improvement in their abilities of teamwork, independent learning, and time management were repeatedly mentioned. In addition, the students admitted that the training facilitated them to fully understand dentistry and dental technology and to draw better blueprints for their future careers.

### Survey 2: Conducted 1 Year After the Course

#### Questionnaires Collection

A total of 22 in 29 dental technology students from the same class provided responses (response rate=approximately 76%), among which 11 students had undertaken the Project 35 training, and the remaining 11 students had not participated in the training. The results are summarized in Table 1. The internal consistency coefficients indicated a highly acceptable reliability of the questionnaire (Cronbach α=0.89). The Kaiser-Meyer-Olkin value of 0.68 indicated an acceptable score with a significant Bartlett’s test of sphericity (*P*<.001; Table 1).

**Table 1:** Comparative analysis of the training group and the nontraining group scores in survey 2.

Item		Training group (n=11), mean (SD)	Nontraining group (n=11), mean (SD)	Pooled SD	Cohen *d*	*z* score		*P* value
**Items in questionnaire**								
	I am satisfied with the skills I have acquired during my college life and think they are adequate for my work in my professional field.	4.27 (0.647)	3.45 (0.934)	0.803	1.02	–2.179		.04
	When there is a technical or financial challenge, I am able to organize and use resources to solve it.	4.18 (0.405)	3.27 (0.786)	0.625	1.45	–2.97		.005
	I often come up with unprecedented ideas and want to make them a reality.	4.09 (0.701)	3.27 (0.647)	0.674	1.21	–2.447		.02
	I’m thinking of starting my own business in the future.	4.27 (0.647)	3.73 (0.786)	0.72	0.76	–1.672		.15
	I think it is necessary to master team skills in college life; it helps a lot at work.	4.36 (0.674)	4.09 (1.044)	0.879	0.31	–0.461		.70
	I am comfortable expressing my own opinions in a group, even when I know that other people don’t agree with them.	4.27 (0.467)	3.55 (0.688)	0.588	1.24	–2.589		.03
	I feel comfortable working in a group.	4.09 (0.701)	3.45 (0.688)	0.694	0.92	–1.926		.09
	I can think from the perspective of coworkers when conflicts happen.	4.18 (0.603)	3.55 (0.82)	0.72	0.88	–1.92		.09
	I recognize and understand the value of innovation and entrepreneurship working in the field of dental technology.	4.18 (0.751)	2.09 (0.944)	0.853	2.45	–3.749		<.001
	I am optimistic about the future of the dental technician industry.	4.09 (0.831)	3.09 (1.136)	0.995	1	–2.083		.047
**Grades**								
	Academic grades	56.19 (2.839)	54.43 (2.497)	2.673	0.66	—^a^	.14	
	Capability grades	25.55 (11.146)	11.83 (9.890)	10.537	1.30	—	.006	

^a^Not available.

#### Questionnaires Analyses

In terms of self-evaluation of their personal ability, the students in the training group were significantly more satisfied with the professional skills they acquired in school than the students in the nontraining group (*z*=–2.179, *P*=.04). There were significant differences between the training group and the nontraining group in meeting challenges (*z*=–2.970, *P*=.005) and innovative thinking (*z*=–2.477, *P*=.02). However, there was no significant difference in the entrepreneurial intentions between the training and nontraining groups. The students in the training group were significantly more comfortable with voicing different opinions in the group than those in the nontraining group. Nevertheless, there were no significant differences between the training group and the nontraining group regarding the need for teamwork, initiative for seeking help from teammates, or empathizing with teammates. In terms of professional identity, the recognition of the value of innovation and entrepreneurship in the professional field of dental technology was significantly higher in the training group than in the nontraining group. We also found that the training group was significantly more optimistic about the future of dental technology as an industry compared to the nontraining group (*z*=–2.083, *P*=.047). The students’ grades could be objectively correlated with their abilities. The capability grades were significantly higher in the training group (Cohen *d*=1.30, *P*=.006), while there were no significant differences in the academic grades between the 2 groups of students.

### One-on-One Email Interview

Subjective positive feedback was also evident from the email interviews of 11 trainees from the training group, who felt that their abilities had considerably improved (11/11, 100%). Many believed that they were “excellent in their professional field” (9/11, 81.8%) and that they “have the same respect and status in a team as dentists” (9/11, 81.8%). In the nontraining group, the proportion of positive feedback was lower than in the training group, with fewer participants reporting that they felt that their abilities had considerably improved (2/11, 18.8%), they were “excellent in their professional field” (4/11, 36.4%), or they “have the same respect status in a team as dentists (0/11, 0%). Several individuals (8/11, 72.7%) in the training group were assigned to specialized positions for communicating with dentists in their postgraduation employment and reported that they received high praise and feedback from their employers who stated that they were well suited to interact with dentists to solve problems, as opposed to those in the nontrained group who were not assigned to such specialized positions. A few skills acquisitions were highlighted in the responses. The knowledge and expertise linked to patents that the participants gathered from the investment road show were perceived to be the most practically valuable. Even though they had not initially opted to actively participate in the project, the students in the nontraining group subsequently felt that it was unfair that only some students could benefit from Project 35 training even though they had not initially chosen to actively participate in the project, and they later wished that they had taken part in Project 35 training like students in the training group.

## Discussion

### Principal Findings

Based on PBL concepts and PBS theory, we creatively created the “Project 35” innovation and entrepreneurship training module for the study. After 4 years of use, the validity of Project 35 was validated, setting the stage for a later, more structured IPE course or training module designed for both dental and dental technology students.

This study demonstrated the validity of the Project 35 IPE module for both dental and dental technology students. Those who received Project 35 training believed that it improved their self-perceived competence in subsequent real-world practice. The application of IPE in dental and dental technology education has been described in several studies. Students at the University of Sheffield’s School of Clinical Dentistry were required to complete 18 courses in order to gain proficiency in both majors’ shared learning and knowledge of concepts [36]. Newcastle upon Tyne Dental Hospital carried out research asking second-year trainee dental technicians and third-year undergraduate dental students to have lectures relevant to this course together, further working together to finish complete dentures for a patient [7]. An IPE curriculum was found to facilitate professional role awareness, teamwork, and shared learning upon collaborative task performance with dental students [32,37]. However, establishing an impactful IPE curriculum is a long-term and challenging process. Although these studies indicate the value of an IPE curriculum for dental disciplines, the course design and implementation in these previous studies show some limitations, particularly in terms of the underlying basic educational theory, content selection, learning approaches, and timing and duration of delivery. Existing IPE courses typically lack a theoretical foundation in educational psychology; therefore, they lack robust solutions to the problems of professional stereotypes and hierarchies [38]. In the previously described curricula, while the students worked in a team and completed assigned duties, systematic theoretical IPE knowledge acquisition through a specialized curriculum was not considered. The students were typically placed directly in team-based task situations without being taught how to “work as a team” [39]. In addition, professional knowledge-based IPE curricula were imparted only in the senior years of university education [40]. Building teamwork skills and understanding the role of other specialties at the early stages of undergraduate training could have added advantages in terms of professional knowledge and skill learning throughout professional education [41]. The curriculum content selection was also determined by the entry timing of the IPE curriculum. The microresearch topics would be different annually and the curricular selected are expected to maximize fit with the topics. It can be difficult to present clinical cases directly as IPE cooperation practice tasks for curriculum are restricted to the very early stages of undergraduate study; therefore, they need to be selected carefully [42]. To address these gaps, we designed a novel pedagogical IPE module and examined its validity when implemented in the first 2 years of undergraduate dental and dental technology programs. This training pedagogy was embodied in a well-organized innovation and entrepreneurship training module named Project 35.

Perspectives and attitudinal aspects play a key role in successful teamwork. A perceived disparity in the dental technicians’ position within the dental care team hierarchy can lead to an attitude that not only causes conflict but also presents barriers to valuable feedback from the technician [43]. Project 35 provides dental and dental technology students with early formative collaborative and exploratory experiences that might prevent the internalization of hierarchical attitudes. The exploratory process is instructor-supervised; in addition to providing subject-specific guidance, it also offers emotional support when both parties encounter difficulties or disputes. As a work practice of consultation and communication between the dentist and the dental technician is established early in the undergraduate program, it becomes likely that graduates will continue this experience in their professional lives, consistent with earlier findings [44]. Our study supports this notion and also proposes another benefit. It’s possible that the IPE-trained dental technicians were better able to address issues because they were more capable of independent learning and innovation, had improved communication skills, and were more willing to express their own opinions in a collaborative way. These factors contributed to the fact that they were more likely to be assigned to positions where they directly communicated with dentists to solve problems. It’s also recognized that interprofessional skills such as role clarity, teamwork, and communication could be improved in dental IPE education at North American universities [45]. The skills taught in the scientific research-training phase of Project 35 were thought practical throughout their college study period and promoted the learning of professional knowledge and skills, and the acquired information retrieval skills helped them to self-address knowledge gaps during their learning of professional skills. A study on IPE in geriatric dental medicine also proved the students’ practical skills improvement in specific medical situations [46]. These findings suggest that while experiential learning inherent to professional life may bridge gaps in communication skills, independent learning and innovation skills might benefit from targeted early development.

This study confirmed a high approval rate of the training module by the students in both professions, which did not differ significantly between the 2 professions, indicating that the validity of the training was largely consistent for both groups. The postcomparative study of the dental technology students confirmed that the self-evaluation of the students in the training group was significantly better than that in the nontraining group in terms of professional skills acquisition, coping with challenges, leveraging opportunities, and innovative thinking. This self-evaluation was objectively verified in terms of capability grades, which confirmed the value of such early training in professional skills development. Of note, the proportion of the total number of dental students who volunteered to participate in the training was significantly lower than that of the dental technology students. A plausible reason could be differences in the expectations regarding the nature of future employment between junior dental and dental technology students [47,48]. Dental students typically enter dentistry undergraduate programs after high school and lack clinical and patient exposure in the first year. Possibly, many students may not perceive the relevance of entrepreneurial training at this stage. However, the junior dental technology students may have had a better understanding of the entrepreneurial nature of future work in commercial dental lab settings; thus, they were more likely to be motivated toward entrepreneurial training. This difference suggests a need to better sensitize dental students toward the benefits of innovation and enterprise training and IPE.

Formal degree-based education has gradually replaced the traditional master-apprentice system and vocational education in dental technology education [49]. This is beneficial considering the rapid technical advancements in dentistry since these must be accompanied by comparable upgrading in professional training and capacity [50]. However, dental technology students receiving a formal education degree may internalize stereotypes about the work and social status of dental technicians from the beginning of their undergraduate study, which can easily lead to a negative bias and lowered intrinsic motivation [51,52]. Positive behavioral approaches can support overcoming such challenging schemes and behaviors [53]. Our findings show that the trained group of dental technology students was more optimistic about the future of dental technology as an industry compared to the nontraining group, demonstrating the validity and promise of PBS as a theory underlying the curriculum.

The lack of objective evaluation of the extent to which IPE enhanced the professional skills of dental and dental technician students is a major limitation of this study. The survey conducted 1 year after the course only addressed the dental technology students, and the training group voluntarily chose to participate, possibly reflecting self-selection bias. Dental students were not included in the second survey, mainly owing to the small percentage of dental students who volunteered for the training and resource limitations. Recruiting and training new instructors to expand the Project 35 training into a formal curriculum is the next step in this direction. The small sample size is another major limitation of this study. Though one-on-one interviews with students were obtained, it is not reasonable to draw robust conclusions from them. Individual responses were mainly positive about the program, but they must be considered only as subjective indicators for the continuation and improvement of the program. Furthermore, the Project 35 training was conducted solely at 1 center. The specific curricular design, scheduling, and participant recruitment may introduce biases that could affect the results. Nevertheless, the implementation of the Project 35 training at this single center could serve as a preliminary reference for future training initiatives at other centers.

Despite these limitations, the present findings indicate that Project 35 training enables teamwork skill acquisition at an early professional stage and is beneficial for the future health of the dental industry with regard to the effective navigation of roles and responsibilities, trust, and mutual respect. Our study demonstrated the potential application of PBS theory in IPE and proposed an innovation and entrepreneurship education curriculum with PBL as a training framework for early IPE for dental and dental technology students. This training framework has the potential for wider application, and further experiments should be carried out to demonstrate its validity.

### Conclusion

Excellent cooperation between dentists and dental technicians is imperative to optimize patient care and experience. Here, we report a novel innovation and entrepreneurship IPE training module designed for undergraduates in dental surgery and dental technology in China and demonstrate its validity. The major limitations of our study are the small sample size and the differences between the broader training goals and the measured outcomes, so the wider implications of our program have not yet been established. However, the overall validity of the program is evident from the self-reported improvements in interdisciplinary cooperation, personal ability, and professional growth among those trained. Future research incorporating a larger sample size, better research designs, and diverse outcomes measured at the pretest, midtest, and posttest stages should be used for the optimization of this IPE curriculum to inform dental education policies.

## Appendix

Multimedia Appendix 1 Microresearch topics.

Multimedia Appendix 2 Recruitment procedure.

Multimedia Appendix 3 Questionnaire 1: Perceptions of participants on Project 35.

Multimedia Appendix 4 Questionnaire 2: Perceptions of graduates in dental technology.

Multimedia Appendix 5 One-on-one email interview.

Multimedia Appendix 6 Ethical approval documents.

Multimedia Appendix 7 Checklist for Reporting Results of Internet E-Surveys (CHERRIES).

Multimedia Appendix 8 Comparative analysis of the scale scores of dental and dental technology students.

## Abbreviations

BARRY: Brave, As a team, Responsible, Realistic, and Young
CHERRIES: Checklist for Reporting Results of Internet E-Surveys
IPE: interprofessional education
PBL: project-based learning
PBS: positive behavior support

## Data Availability

The datasets generated during and/or analyzed during this study are available from the corresponding author upon reasonable request.
