# Supplementary material for: Development of an Interprofessional Education Project in Dentistry Based on the Positive Behavior Support Theory: Pilot Curriculum Development and Validation Study
Source: JMIR Form Res. 2024 Nov 11;8:e50389. doi: 10.2196/50389 (PMC11589498; doi:10.2196/50389)
Supplement: Multimedia Appendix 1 [file formative_v8i1e50389_app1.docx]

**Micro-research topics**

1. A nano-micro alternating multilayer scaffold loading with BMP4 for the reconstruction and regeneration of skull defects
2. Full cycle management platform for pediatric orthodontics
3. A virtual simulation-based teaching and examination system for oral-related antecedent skills training and clinical operation skills
4. Sequential cartoon assisted therapy services in pediatric dentistry
5. Online communication platform for dentists and dental technicians
