# Supplementary material for: Development of an Interprofessional Education Project in Dentistry Based on the Positive Behavior Support Theory: Pilot Curriculum Development and Validation Study
Source: JMIR Form Res. 2024 Nov 11;8:e50389. doi: 10.2196/50389 (PMC11589498; doi:10.2196/50389)
Supplement: Multimedia Appendix 2 [file formative_v8i1e50389_app2.docx]

**Recruitment Procedure**

**Step 1. Send notification of registration** We write the recruitment notice in Chinese and review it by two students in second-year student and a principal investigator, to avoid ambiguity. The context of recruitment notice is displayed as follow:

*Dear Potential Participants:*

*Hello, thank you for spending time to read this notice.*

*There is a new curriculum designed for first-year dental and dental technology. This curriculum is innovative and unprecedented, aiming at improving your abilities to handle some entrepreneur and innovation activities, including self-learning, teamwork, communication and speech. All participants are encouraged to finish a project as a final duty. If you decide to get involved in this curriculum, please get ready for more burden and less leisure time in future two years.*

*If you would like to participate, please send an email to the assigned email address, which should include your basic information (name and student ID) and resume.*

*We look for your participation. Thank you!*

*Project 35*

**Step 2.** **Collect student’s information** Read emails sent by students and collecting their information in an Excel table.

**Step 3. Confirm students’ presence** Send an email to the students’ email address to reconfirm their participation in Project 35 and notify them of the time of the first lesson.

**Step 4. Final check** Count attendance in the first lesson and update the Excel table.
