# Supplementary material for: Development of an Interprofessional Education Project in Dentistry Based on the Positive Behavior Support Theory: Pilot Curriculum Development and Validation Study
Source: JMIR Form Res. 2024 Nov 11;8:e50389. doi: 10.2196/50389 (PMC11589498; doi:10.2196/50389)
Supplement: Multimedia Appendix 3 [file formative_v8i1e50389_app3.docx]

**Questionnaire 1: Perceptions of participants on Project 35**

*Dear Participants:*

*Hello, thank you very much for taking out your valuable time, we sincerely invite you to fill in this questionnaire to facilitate the understanding of your views on Project 35, to promote the continuous improvement of it. We also hope that you will agree to provide us with the data of the questionnaire that you have filled in for the purpose of analyses. This questionnaire is only for the participants of the School of Stomatology, Chongqing Medical University, and the participants do not bear all legal responsibilities arising from the questionnaire being quoted from the date of this statement, which is hereby declared.*

*Project 35*

1. Who are you? (name, grades, and majors)

________________________

2. Duration of participation

○ One year ○ Two years ○ Three years ○ Four years

3. Knew about innovation and entrepreneurship before training

○ Strongly Disagree ○ Disagree ○ Neutral ○ Agree ○Strongly Agree

4. Achieved self-improvement

○ Strongly Disagree ○ Disagree ○ Neutral ○ Agree ○Strongly Agree

5. Be involved in interprofessional education

○ Strongly Disagree ○ Disagree ○ Neutral ○ Agree ○Strongly Agree

6. Know more about the other major(s)

○ Strongly Disagree ○ Disagree ○ Neutral ○ Agree ○Strongly Agree

7. Improved active learning ability

○ Strongly Disagree ○ Disagree ○ Neutral ○ Agree ○Strongly Agree

8. Improved relearning ability

○ Strongly Disagree ○ Disagree ○ Neutral ○ Agree ○Strongly Agree

9. Learn stress management

○ Strongly Disagree ○ Disagree ○ Neutral ○ Agree ○Strongly Agree

10. Improved coping with frustration

○ Strongly Disagree ○ Disagree ○ Neutral ○ Agree ○Strongly Agree

9. Improved lateral thinking skills

○ Strongly Disagree ○ Disagree ○ Neutral ○ Agree ○Strongly Agree

10. Improved empathy

○ Strongly Disagree ○ Disagree ○ Neutral ○ Agree ○Strongly Agree

11. Improved teamwork skills

○ Strongly Disagree ○ Disagree ○ Neutral ○ Agree ○Strongly Agree

12. The training is positive and beneficial

○ Strongly Disagree ○ Disagree ○ Neutral ○ Agree ○Strongly Agree

13. Please describe what you gained from Project 35

________________________
