# Supplementary material for: Development of an Interprofessional Education Project in Dentistry Based on the Positive Behavior Support Theory: Pilot Curriculum Development and Validation Study
Source: JMIR Form Res. 2024 Nov 11;8:e50389. doi: 10.2196/50389 (PMC11589498; doi:10.2196/50389)
Supplement: Multimedia Appendix 4 [file formative_v8i1e50389_app4.docx]

**Questionnaire 2: Perceptions of graduates in dental technology**

*Dear Graduates in dental technology:*

*Hello, thank you very much for taking out your valuable time, we sincerely invite you to fill in this questionnaire. We also hope that you will agree to provide us with the data of the questionnaire that you have filled in for the purpose of analyses. This questionnaire is only for graduates in dental technology of the School of Stomatology, Chongqing Medical University, and there are no risk of bearing all legal responsibilities arising from the questionnaire being quoted from the date of this statement, which is hereby declared.*

*Project 35*

1. Who are you? (name, grades, and majors)

________________________

2. Have you ever been involved in Project 35

○ Yes ○ No

3. I am satisfied with the skills I have acquired during my college life and think they are adequate for my work in my professional field.

○ Strongly Disagree ○ Disagree ○ Neutral ○ Agree ○Strongly Agree

4. When there is a technical or financial challenge, I am able to organize and use resources to solve it.

○ Strongly Disagree ○ Disagree ○ Neutral ○ Agree ○Strongly Agree

5. I often come up with unprecedented ideas and want to make them a reality.

○ Strongly Disagree ○ Disagree ○ Neutral ○ Agree ○Strongly Agree

6. I’m thinking of starting my own business in the future.

○ Strongly Disagree ○ Disagree ○ Neutral ○ Agree ○Strongly Agree

7. I think it is necessary to master team skills in college life; it helps a lot at work.

○ Strongly Disagree ○ Disagree ○ Neutral ○ Agree ○Strongly Agree

8. I am comfortable expressing my own opinions in a group, even when I know that other people don’t agree with them.

○ Strongly Disagree ○ Disagree ○ Neutral ○ Agree ○Strongly Agree

9. I feel comfortable working in a group.

○ Strongly Disagree ○ Disagree ○ Neutral ○ Agree ○Strongly Agree

10. I can think from the perspective of coworkers when conflicts happen.

○ Strongly Disagree ○ Disagree ○ Neutral ○ Agree ○Strongly Agree

11. I recognize and understand the value of innovation and entrepreneurship working in the field of dental technology.

○ Strongly Disagree ○ Disagree ○ Neutral ○ Agree ○Strongly Agree

12. I am optimistic about the future of the dental technician industry.

○ Strongly Disagree ○ Disagree ○ Neutral ○ Agree ○Strongly Agree
