# Supplementary material for: Development of an Interprofessional Education Project in Dentistry Based on the Positive Behavior Support Theory: Pilot Curriculum Development and Validation Study
Source: JMIR Form Res. 2024 Nov 11;8:e50389. doi: 10.2196/50389 (PMC11589498; doi:10.2196/50389)
Supplement: Multimedia Appendix 5 [file formative_v8i1e50389_app5.docx]

**One on One Email Interview**

*Dear Dental Technology Graduates:*

*Hello, thank you very much for taking out your valuable time, we sincerely invite you to answer these questions provided in the email, where you are encouraged to share some opinions and feelings about university life and Project 35 (maybe you didn’t get involved in Project 35).We also hope that you will agree to provide us with the answers that you have filled in for the purpose of analyses. This questionnaire is only for the students in the School of Stomatology, Chongqing Medical University, and the students do not bear all legal responsibilities arising from the interview being quoted from the answers of this statement, which is hereby declared.*

*Project 35*

1. Who are you? (name, grades, and majors)

________________________

2. Do you think your skills were promoted in university period?

________________________

If the answer is yes, please answer the following questions:

2.1 How did you acquire the skills?

________________________

2.2 What skills do you think useful?

________________________

2.3 How these skills benefit you?

________________________

3. What do you think of your performance in your professional field?

________________________

4. What do you think of working with dentists? Do you think you share the same respect and status as dentists?

________________________

5. What’s your role in your present employment? How do you like it? ________________________

6. Have you ever regretted participating in Project 35? (For graduates in training group)

________________________

7. Have you ever regretted not participating in Project 35? (For graduates in nontraining group)

________________________
