# Supplementary material for: Development of an Interprofessional Education Project in Dentistry Based on the Positive Behavior Support Theory: Pilot Curriculum Development and Validation Study
Source: JMIR Form Res. 2024 Nov 11;8:e50389. doi: 10.2196/50389 (PMC11589498; doi:10.2196/50389)
Supplement: Multimedia Appendix 6 [file formative_v8i1e50389_app6.pdf]

NO: 2022（LSNo.030）

Research Ethics Committee
The Affiliated Hospital of Stomatology , Chongqing Medical University

Resolution Paper of Scientific Experiments Ethical Review

Experiment title: Innovative Interprofessional Education (IPE) in Dentistry: Project 35

Correlative content: B
(A. Animal experiment or specimens. B. Specimens taken from human body. C. Embryonic stem cells)

Dear Doc. MengWei Pang

The Research Ethics Committee reviewed your research project titled above and made the resolution as the following item 1. :

1. The experiment involved ethical part conforms to the scientific experiment ethical requirements, it is agreed to implement the experiment.

2. The experiment involved ethical part basically conforms to the scientific experiment ethical requirements, but the following relevant contents should be modified:

3. The experiment involved ethical part does not conform to the scientific experiment ethical requirements, it is not agreed to implement the experiment.

REC Reference Number: CQHS-REC-2022（LSNo.030）
(Please quote this ref # on all correspondence)

Research Ethics Committee
The Affiliated Hospital of Stomatology
Chongqing Medical University

Date of Issue : 2022.4.15
