# Supplementary material for: Development of an Interprofessional Education Project in Dentistry Based on the Positive Behavior Support Theory: Pilot Curriculum Development and Validation Study
Source: JMIR Form Res. 2024 Nov 11;8:e50389. doi: 10.2196/50389 (PMC11589498; doi:10.2196/50389)
Supplement: Multimedia Appendix 8 [file formative_v8i1e50389_app8.docx]

|  | **Mean** | | **SD** | **Pooled SD** | **Cohen’s D** | ***P*-value** |
| --- | --- | --- | --- | --- | --- | --- |
| **Knew about** **innovation and entrepreneurship before training** | | | | | | |
| Dental students (*n*=24) | 2.58 | | 0.929 | 1.003 | -0.07 | .82 |
| Dental technology students (*n*=23) | 2.65 | | 1.071 |  |  |  |
| **Achieved self-improvement** | | | | | | |
| Dental students (*n*=24) | 4.63 | | 0.495 | 0.501 | 0.12 | .68 |
| Dental technology students (*n*=23) | 4.57 | | 0.507 |  |  |  |
| **Be involved in interprofessional education** | | | | | | |
| Dental students (*n*=24) | 4.13 | | 0.797 | 0.834 | -0.01 | .98 |
| Dental technology students (*n*=23) | 4.13 | | 0.869 |  |  |  |
| **Know more about the other major(s)** | | | | | | |
| Dental students (*n*=24) | 4.33 | | 0.702 | 0.730 | 0.28 | .35 |
| Dental technology students (*n*=23) | 4.13 | | 0.757 |  |  |  |
| **Improved active learning ability** | | | | | | |
| Dental students (*n*=24) | 4.54 | | 0.509 | 0.620 | -0.18 | .54 |
| Dental technology students (*n*=23) | 4.65 | | 0.714 |  |  |  |
| **Improved relearning ability** | | | | | | |
| Dental students (*n*=24) | 4.54 | | 0.588 | 0.591 | 0.03 | .91 |
| Dental technology students (*n*=23) | 4.52 | | 0.593 |  |  |  |
| **Learn stress management** | | | | | | |
| Dental students (*n*=24) | 4.38 | | 0.711 | 0.752 | -0.14 | .64 |
| Dental technology students (*n*=23) | 4.48 | | 0.790 |  |  |  |
| **Improved coping with frustration** | | | | | | |
| Dental students (*n*=24) | 4.29 | | 0.751 | 0.751 | 0.04 | .89 |
| Dental technology students (*n*=23) | 4.26 | | 0.752 |  |  |  |
| **Improved lateral thinking skills** | | | | | | |
| Dental students (*n*=24) | 4.42 | 0.654 | | 0.685 | 0.10 | .73 |
| Dental technology students (*n*=23) | 4.35 | 0.714 | |  |  |  |
| **Improved empathy** | | | | | | |
| Dental students (*n*=24) | 4.46 | 0.658 | | 0.662 | -0.03 | .92 |
| Dental technology students (*n*=23) | 4.48 | 0.665 | |  |  |  |
| **Improved teamwork skills** | | | | | | |
| Dental students (*n*=24) | 4.46 | 0.588 | | 0.589 | -0.18 | .54 |
| Dental technology students (*n*=23) | 4.57 | 0.590 | |  |  |  |
| **The training is positive and beneficial** | | | | | | |
| Dental students (*n*=24) | 4.54 | 0.658 | | 0.639 | -0.31 | .30 |
| Dental technology students (*n*=23) | 4.74 | 0.619 | |  |  |  |

Multimedia Appendix 8. Comparative analysis of the scale scores of dental and dental technology students.
